# Relationship between nutrition knowledge and dietary intake among primary school children in Japan: Combined effect of children's and their guardians' knowledge

**DOI:** 10.1016/j.je.2016.09.014

**Published:** 2017-05-30

**Authors:** Keiko Asakura, Hidemi Todoriki, Satoshi Sasaki

**Affiliations:** a Department of Environmental and Occupational Health, School of Medicine, Toho University, Tokyo, Japan; b Department of Social and Preventive Epidemiology, School of Public Health, The University of Tokyo, Tokyo, Japan; c Tropical Biosphere Research Center, University of the Ryukyus, Okinawa, Japan

**Keywords:** Nutrition knowledge questionnaire, Primary school child, Guardian, Dietary intake, Japan

## Abstract

**Background:**

Improving the dietary habits of children is important to decrease the future burden of noncommunicable diseases. While various food education programs have been implemented worldwide, evaluation of nutrition knowledge is difficult, even at baseline. Further, the relationship between nutrition knowledge and dietary intake has not been clarified in non-western countries.

**Methods:**

After developing nutrition knowledge questionnaires for Japanese primary school children and adults, we examined whether higher nutrition knowledge of children and their guardians was associated with better dietary intake in children. A total of 1210 children in four public primary schools and 319 guardians were included in this cross-sectional study.

**Results:**

Nutrition knowledge questionnaires were developed for children in lower and higher grades and adults. Higher nutrition knowledge of the children was significantly associated with higher vegetable intake (*p* for trend = 0.024 for boys and <0.0001 for girls in lower grades, <0.0001 for boys and 0.020 for girls in higher grades). Higher nutrition knowledge of the guardians was also associated with higher vegetable intake, except for boys in higher grades. The relationship between guardians' nutrition knowledge and intake of staple foods and fruits in children differed by children's sex.

**Conclusions:**

We developed nutrition knowledge questionnaires for Japanese children and adults and identified a relationship between higher nutrition knowledge and healthier dietary habits. The child's own nutrition knowledge of dietary intake might be as important as that of the guardian for some foods. Sex differences in the effect of nutrition knowledge should receive greater attention in food education.

## Highlights

• Nutrition knowledge questionnaires were developed for Japanese children and adults.
• Higher nutrition knowledge was associated with healthier dietary habits in children.
• Both children's and guardians' nutrition knowledge affected food intakes in children.
• Sex differences in the effect of nutrition knowledge were suggested.

## Introduction

Dietary habits are established in childhood and track into adulthood.^1^ Since the risk of many noncommunicable diseases is closely related with dietary habits, various dietary education programs aimed at establishing healthy dietary habits in children have been implemented worldwide.^2^, ^3^

“Shokuiku” is a Japanese word meaning food education or dietary education.^4^ Following enactment of the Basic Law on Shokuiku (law for food education promotion) in 2005, several education programs were implemented in Japan. To date, however, no Japanese food education program has been quantitatively evaluated for effectiveness. Overseas, in contrast, a number of questionnaires to evaluate nutrition knowledge have been developed, mostly for adult use.^5^, ^6^, ^7^, ^8^ For instance, Parmenter and Wardle established a questionnaire to comprehensively investigate nutrition knowledge in the United Kingdom and confirmed its reliability and validity,^5^ and Wardle et al. reported that knowledge evaluated using this questionnaire was significantly associated with healthy eating, namely with greater fruit and vegetables intake and less fat intake.^9^ The questionnaire has also been employed by Australian^10^ and Turkish^11^ groups. A few age-specific questionnaires have also been developed for children and adolescents.^7^, ^8^ Grosso et al. showed that improving nutrition knowledge in children and young adolescents may have led them to adopt better dietary habits.^12^

In their review, Spronk et al. showed that approximately 70% of studies conducted in adult populations reported significant but weak positive associations between higher nutrition knowledge and better dietary intake.^13^ However, they also stated that methods used to assess nutrition knowledge and dietary intake were heterogeneous and that a full understanding of the relationship required well-designed studies using validated methodologies. Thus, despite the key importance of nutrition knowledge in establishing better dietary habits and health, further studies are still necessary.

Here, we developed three types of age-specific questionnaires for a Japanese population, two for primary school children in lower and higher grades and a third for adults to evaluate nutrition knowledge quantitatively. We then used these questionnaires and a validated diet history questionnaire in primary school children and their guardians to describe the level of nutrition knowledge and examine the relationship between nutrition knowledge and dietary intake.

## Methods

### Study participants

A food education program was newly implemented as part of the study curriculum for primary school children from May 2014 in Yaese, Okinawa Prefecture, Japan. The town has four primary schools, and all 1944 pupils participated in the food education program. These children and their guardians, who mainly prepare meals for the children, were recruited to the study in April 2014, before the beginning of the education program. The total number of children who agreed to be included in the study as participants was 1210, of whom 623 were in lower grades (1–3) and 587 were in higher grades (4–6) (participation rate, 1210/1928 = 62.8%; the questionnaires were not distributed to 16 pupils in a special class for handicapped children). A total of 319 guardians agreed to participate in the study (23.1%, taking the total number of families [1382] in the schools as denominator in consideration of siblings). No other exclusion criteria were set, on the basis that the collaborating schools were public and that all pupils should accordingly be educated equally. This study was approved by the Ethics Committee of the University of Tokyo, Faculty of Medicine (pilot study: approval no. 10315, approval date November 1, 2013; main research: approval No. 10418, approval date March 3, 2014). Written informed consent was obtained from the guardians of all participating children and all participating guardians.

### Study outline

The children and guardians each completed two questionnaires, a nutrition knowledge questionnaire and a diet history questionnaire. Questionnaires for the children were distributed through the primary schools in May 2014, before the beginning of the food education program. The children were asked to complete the nutrition knowledge questionnaire at home without their guardian's advice. The guardians received their questionnaires through the schools and answered them from June to September 2014. All questionnaires were collected by the study center at University of the Ryukyus and checked by researchers, who confirmed any unclear points with the participants through the schools.

### Nutrition knowledge questionnaire for primary school children and adults

The framework of the nutrition knowledge questionnaire in this study was based on the questionnaire for adults developed by Parmenter and Wardle.^5^ They have showed good validity and reliability of their questionnaire.^5^ It has five main sections: a) the understanding of terms; b) awareness of dietary recommendations; c) which foods contain which nutrients; d) using the information to make dietary choices; and e) awareness of diet–disease associations. The questionnaire for Japanese adults established for our study also included five sections: 1) knowledge about foods as nutrient sources (corresponding to Section c of Parmenter's questionnaire); 2) physiological function of nutrients in the human body (corresponding to Sections a and d); 3) awareness of dietary recommendations (corresponding to Section b); 4) relationship between nutrients and health outcomes (corresponding to Section e); and 5) other questions relating to the dietary behavior or background factors of subjects (corresponding to Section d). For primary-school children, two questionnaires were developed, one used in children in grades one to three (lower grader) and the second in those in grades four to six (higher grader). The sections included in the questionnaire for children were Sections 1, 2, 4, and 5. Since it was difficult to ask lower-grade children about the details of nutrients, a new section asking them to categorize foods based on nutritional similarity was added to support Sections 1 and 2. Section 5 in our questionnaire was not used to calculate the total knowledge score in children in higher grades or adults, because the right answer for each question differed among respondents according to differences in individual dietary intake and health condition.

The items (questions) in the questionnaire were determined as follows. First, we searched PubMed for papers written in English that included a full description of all items in a nutrition knowledge questionnaire. References from relevant articles were manually searched. No Japanese paper on nutrition knowledge questionnaires was found. We then selected those questionnaires that included questions about multiple nutrients (one was just for salt intake^14^) and those established for use in developed countries. The number of papers selected was 23 (four papers for lower graders,^7^, ^15^, ^16^, ^17^ 11 for higher graders,^8^, ^14^, ^18^, ^19^, ^20^, ^21^, ^22^, ^23^, ^24^, ^25^, ^26^ and eight for adults.^5^, ^6^, ^9^, ^27^, ^28^, ^29^, ^30^, ^31^ Items in most papers could be applied to multiple age categories. All questions in these questionnaires were then translated into Japanese. The list of questions included 116 items for lower graders, 168 for higher graders, and 138 for adults. After categorizing the listed questions by content similarity and administration method, different types of questions were selected as candidates for our questionnaires considering content validity. Since all questionnaires were established in other countries, foods, nutrients, and dietary behaviors had to be reconsidered to match Japanese food culture. We selected foods that are frequently consumed in Japan and easily recognized by children, with consideration to nutritional characteristics.^32^ Also, we included nutrients known to be deficient or excess in the Japanese population based on the report of the National Health and Nutrition Survey in Japan^33^ as question items. In the questionnaires for school children, wording was carefully selected for reading level based on the study courses and textbooks for food education established by the Ministry of Education, Culture, Sports, Science and Technology, Japan.^34^, ^35^

The temporary questionnaires used in the pilot study included 46 items for lower graders, 70 items for higher graders, and 145 items for adults. School child and guardian pairs were recruited using convenience sampling (19 lower graders, 25 higher graders, and 41 guardians; three children had siblings) from various areas in Japan (Tokyo, Aichi, Hyogo, and Nagasaki Prefectures) and asked to complete the questionnaire. Face validity, time required for response, and comprehensibility of questions and terms were also confirmed by obtaining comments from the respondents. We then calculated the percentage of correct answers for each question, and basically retained those which 30–90% of respondents answered correctly.^5^ Items that met the criteria but overlapped with other questions were deleted, while those that did not meet the criteria but were essential to evaluate a certain aspect of nutrition knowledge were retained. For example, questions about major nutrients in staple foods, such as rice and bread, and those about sugary foods were retained. After this process, the final questionnaires included 32 items for lower graders (6 pages), 62 items for higher graders (7 pages), and 119 items (8 pages) for adults. Of these, the percentage of correct answers in lower graders was calculated from 26 items after the exclusion of items in Section 5. Calculations for higher graders and guardians were done similarly using 27 and 84 items, respectively. Content validity of the final questionnaire was rechecked independently by the research team, including the authors, research dietitians in the field of applied nutrition, and on-site dietitians working in the research area, before the main study. All items in the three questionnaires that were used to determine the nutrition knowledge score are shown in eTable 1.

### Brief-type, self-administered diet history questionnaire (BDHQ) and brief-type, self-administered diet history questionnaire for school children and adolescents (BDHQ15y)

The BDHQ is a four-page fixed-portion questionnaire that asks about the consumption frequency of selected foods commonly consumed in Japan, general dietary behavior, and usual cooking methods to estimate the dietary intake of 58 food and beverage items during the preceding month. The BDHQ has been validated^36^, ^37^ and used in several epidemiologic studies.^38^, ^39^ It was used for guardians, but their dietary intake data were not analyzed in this study.

Nutrient and food intake of the children was estimated using the BDHQ15y. The children were asked to answer this questionnaire with their guardians to ensure its good validity.^40^ The BDHQ15y is a four-page questionnaire developed based on the self-administered diet history questionnaire (the 16-page comprehensive type)^36^, ^37^, ^41^ and the BDHQ for adults and can be used for school children and adolescents aged 6–18 years. The basic structure of the BDHQ15y is closely similar to that of the BDHQ for adults but excludes one section about alcoholic beverages. Estimates of daily intake for foods (67 items in total), energy, and selected nutrients were calculated using an ad hoc computer algorithm for the BDHQ15y based on the Standard Tables of Food Composition in Japan.^32^ The validity of the BDHQ15y for selected fatty acids and carotenoids using biomarkers (erythrocyte fatty acids and serum carotenoids) as a gold standard has been reported elsewhere.^41^

### Other measurements

Body height and weight of the children were measured as part of a routine health checkup by school nurses at each school in April 2014. Heights and weights of the guardians were self-reported in the questionnaires. Background and lifestyle information of the participants was collected using the nutrition knowledge questionnaire.

### Data analysis

Among all participants of the study, six lower graders, eight higher graders, and three guardians did not answer the BDHQ or BDHQ15y and were excluded from analysis. In addition, participating children whose energy intake estimated using the BDHQ15y was not between ≥0.5 times the estimated energy requirement (EER) for people with the lowest physical activity level (EER I)^42^ and <1.5 times the EER for those with the highest physical activity level (EER III)^42^ were also excluded (25 lower and 21 higher graders). The final number of analyzed subjects was 592 for lower graders and 558 for higher graders. Since we did not use the dietary intake data of the guardians, we did not exclude those whose response to the BDHQ was inadequate, and the number of analyzed guardians remained at 316. To examine the effect of guardians' nutrition knowledge on children's dietary intake, the children's dataset was merged with the guardians' dataset. This left 219 lower grader–guardian pairs and 205 higher grader–guardian pairs in the merged dataset.

We calculated the percentage of correct answers for each section in the three types of age-specific nutrition knowledge questionnaires. The internal consistency between sections in each questionnaire was evaluated using Cronbach's alpha. The internal consistency between questions could not be confirmed using the same index, because answers for many questions were categorical (yes/no response) rather than scaled.

Food intake in the children was then described in four levels (quartiles) of the children's nutrition knowledge groups (low, medium–low, medium–high, and high). Trends of association between food intake and knowledge quartile were examined using a linear regression model, which included food intake as the dependent variable and knowledge quartile and the child's grade as independent variables. The *P* values for linear trends were calculated by assigning each knowledge quartile a score (low = 1, medium–low = 2, medium–high = 3, and high = 4). All analyses were performed separately for lower and higher graders and for boys and girls. Food intakes in the analysis were energy-adjusted and presented in weight (g) per 1000 kcal of energy intake. Next, food intake in the child was described in three levels (tertiles) of the guardians' nutrition knowledge groups, and the difference between groups was evaluated by the same method as that for the children's knowledge. Finally, the combined effect of the child and guardians' knowledge on the selected food intake of the child was examined. Linear regression model including children's food intake as a dependent variable was used. Effect of children's sex, grade, and guardians' income was adjusted in model 1. In addition, existence of effect modification by children's sex for the relationship between food intake and nutrition knowledge was assessed (model 2 and model 3). Model 2 included an interaction term between children's knowledge and children's sex, and model 3 included that between guardians' knowledge and children's sex.

All analyses were performed with Statistical Analysis System (SAS) version 9.4 software (SAS Institute, Cary, NC, USA). Statistical tests were two-sided, and *P* values of <0.05 were considered statistically significant.

## Results

Subject characteristics are shown in Table 1. The structure, number and example of questions, and percentage of correct answers in each section are shown in Table 2. The mean percentage of correct answers in each questionnaire was 77.4% among lower graders, 68.5% among higher graders, and 70.1% among guardians. Cronbach's alphas were 0.66 (lower graders), 0.76 (higher graders), and 0.59 (guardians). Since exclusion of the section about awareness of dietary recommendations improved the Cronbach's alpha of the guardians' questionnaire to 0.69, this section was not included in later analysis.

**Table 1:** Characteristics of subjects.

Variables	Category	*n* (%) or mean, SD		
		Grades 1–3 (*n* = 592)	Grade 4–6 (*n* = 558)	Guardians (*n* = 316)
School^a^	A	325 (54.9)	297 (53.2)	159 (50.3)
	B	129 (21.8)	123 (22.0)	77 (24.4)
	C	95 (16.1)	98 (17.6)	52 (16.5)
	D	43 (7.3)	40 (7.2)	28 (8.9)

Children's grade^b^	1	205 (34.6)	–	88 (27.9)
	2	204 (34.5)	–	78 (24.7)
	3	183 (30.9)	–	49 (15.5)
	4	–	199 (35.7)	43 (3.6)
	5	–	197 (35.3)	37 (11.7)
	6	–	162 (29.0)	21 (6.7)

Age, years	6	173 (29.2)	–	–
	7	212 (35.8)	–	–
	8	193 (32.6)	–	–
	9	14 (2.4)	171 (30.7)	–
	10	–	200 (35.8)	–
	11	–	161 (28.9)	–
	12	–	26 (4.7)	–

	Mean, SD	–	–	39.5, 6.4
	20–29	–	–	8 (2.5)
	30–39	–	–	162 (51.3)
	40–49	–	–	133 (42.1)
	50–59	–	–	9 (2.9)
	60–	–	–	4 (1.3)

Sex	Boy, men	287 (48.5)	292 (52.3)	25 (7.9)
	Girl, women	305 (51.5)	266 (47.7)	291 (92.1)

Height, cm	Mean, SD	120.8 (6.8)	138.7, 8.0	157.4, 6.9
Weight, kg	Mean, SD	23.5 (4.4)	34.2, 8.0	56, 10.1
BMI	Mean, SD	–	–	22.6, 3.6

Energy intake, kcal/day	Mean, SD	1614 (455)	1980, 672	–

Household income, yen	<3 million	–	–	92 (29.1)
	3 to <6 million	–	–	140 (44.3)
	6 to <9 million	–	–	51 (16.1)
	≥9 million	–	–	21 (6.6)
	Missing	–	–	12 (3.8)

a The numbers of all enrolled pupils in each primary school were 955 for school A, 512 for school B, 334 for school C, and 143 for school D, respectively.

b If guardians have two or more children, they are classified into the lowest grade in their children.

**Table 2:** Structure, internal consistency, and percentage of correct answers of the nutrition knowledge questionnaire for Japanese school children and their guardians.

	Knowledge section	Number of questions^a^	Examples of questions	Internal consistency (Cronbach's alpha)^b^	Percentage of correct answers, mean (SD)
NKQ for lower grader (for pupils in grade 1–3)	Total	26		0.66	77.4 (11.9)
	Categorization of foods based on nutritional similarity	6	Circle the item number of one food which is different from other foods in a group of four foods (answer choices: cabbage, cucumber, Japanese radish, rice)		79.8 (21.4)
	Knowledge about foods as nutrient sources	10	Which food contains more salt? (answer choices: pork, sausage)		72.2 (20.3)
	Physiological function of nutrients in human body	3	Choose two foods which mainly work as energy (heat and power) in the human body (answer choices: rice, lettuce, egg, spaghetti, string beans)		71.0 (17.0)
	Relationship between nutrients and health outcomes	1	Choose all foods which should be consumed more to become healthy (e.g. of answer choices: carrot, french fries, milk, chocolate)		86.0 (14.6)
	Others 1 (e.g. dietary behavior, food choice)	6	It is the best for you to flavor foods as you like (answer choices: correct, wrong).		85.7 (16.5)
	Others 2 (e.g. background of subjects)	(6)	Are you a boy or a girl?	–	–

NKQ for higher grader (for pupils in grade 4–6)	Total	27		0.76	68.5 (15.0)
	Knowledge about foods as nutrient sources	7	Circle the item numbers of three foods which should be consumed when you need calcium (answer choices: buckwheat noodles, tuna, cheese, potatoes, Shirasuboshi,^c^ soybeans)		72.1 (13.8)
	Physiological function of nutrients in human body	10	The nutrient which mainly constitutes blood and muscles is carbohydrates (answer choices: correct, wrong, not sure).		59.6 (25.4)
	Relationship between nutrients and health outcomes	10	Excess energy intake from meals does not cause health problems in children (answer choices: correct, wrong, not sure).		66.5 (25.1)
	Others 1 (e.g. dietary behavior, communication with families)	(30)	How many times do you have breakfast per week?	–	–
	Others 2 (e.g. background of subjects)	(5)	Are you a boy or a girl?	–	–

NKQ for adults	Total	84		0.59^d^	70.1 (11.0)
	Knowledge about foods as nutrient sources	42	Are foods listed below rich in starch? (cheese, butter, nuts, rice, cornflakes; answer choices: rich, poor, not sure)		74.1 (13.3)
	Physiological function of nutrients in human body	15	Only excess intake of fat and oil causes obesity (answer choices: correct, wrong, not sure).		70.7 (15.8)
	Awareness of dietary recommendations	5	According to the recommendation of the Ministry of Health, Labour and Welfare, how much salt intake (gram per day) is recommended for Japanese adults (more than 18 years old)? (e.g. of answer choices: less than 5 g for men, less than 4.5 g for women)		43.5 (20.5)
	Relationship between nutrients and health outcomes	22	Do you think that risk of a certain cancer can be decreased by actions listed below? (e.g. of actions: consume more dietary fiber; answer choices: decrease, not decrease, not sure)		61.6 (14.3)
	Others 1 (e.g. dietary behavior, communication with families)	(22)	I am careful not to eat too much (answer choices: always do so, do so, rarely do so, never do so).	–	–
	Others 2 (e.g. background of subjects)	(13)	Which school did you graduate at last? Circle an alphabet.		

NKQ, nutrition knowledge questionnaire.

a The number of questions shown in parenthesis was not included in the total number of questions in each questionnaire, because they were not used to calculate percentage of correct answers.

b Internal consistency between the knowledge sections was evaluated by calculating Cronbach's alpha.

c Shirasuboshi, boiled and dried young anchovies.

d Cronbach's alpha for the guardian's nutrition knowledge questionnaire was 0.69 after excluding the section about awareness of dietary recommendation from the calculation. Therefore, this section was excluded from calculation of the percentage of correct answers used in later analysis.

The relationship between the children's nutrition knowledge and their dietary intake is shown in Table 3. A higher knowledge level was significantly associated with a higher vegetable intake. The intake of staple foods was conversely associated with nutrition knowledge in boys (positive association) and girls (negative association) in lower grades. Table 4 summarizes the relationship between the guardians' nutrition knowledge and child's dietary intake. Higher guardian knowledge was significantly associated with higher intake of vegetables in lower graders. Higher fruit intake was significantly or marginally related with higher guardian knowledge only in girls. Last, the combined effect of children's and guardians' nutrition knowledge on the selected food intake of children was examined (Table 5). Because effect modification by children's sex was suspected in the previous analysis, it was also considered using regression models. The analysis showed that higher children's nutrition knowledge and guardians' nutrition knowledge were significantly and independently associated with higher vegetable intake (model 1). No effect modification by children's sex was observed in the relationship between children's nutrition knowledge and their food intakes (model 2). On the other hand, effect modification was observed in the relationship between nutrition knowledge in guardians and intake of staple foods and fruits in children (model 3). Nutrition knowledge in neither children nor guardians was significantly associated with intake of sweets and snacks. Regarding other factors, children's sex was marginally or significantly associated with intake of vegetables and sweets and snacks. Guardians' income was marginally associated with children's fruit intake. The regression models including guardians' educational background instead of guardians' income showed the same results (data not shown in the tables).

**Table 3:** Relationship between the percentage of correct answers for the nutrition knowledge questionnaire in primary school children and their dietary intakes.

Analyzed population	Groups by the percentage of correct answers	Boys					Girls				
		Low	Medium low	Medium high	High	*p* for trend^a^	Low	Medium low	Medium high	High	*p* for trend^a^
Grades 1–3	Number of respondents	67	77	76	67		78	76	75	76	
	% of correct answers	61.1	74.1	82.3	92.6		61.9	73.7	82.0	92.3	
	Food intakes, adjusted mean,^b^ g/1000 kcal										
	Staple foods^c^	236.6	239.2	249.7	257.8	0.038	240.6	243.4	238.4	217.5	0.023
	Rice	181.7	188.3	204.1	212.4	0.0075	189.8	189.1	190.7	169.4	0.10
	Bread	18.2	21.8	15.8	17.1	0.19	19.1	20.8	17.5	19.4	0.72
	Meat	36.3	35.5	32.6	30.3	0.0066	32.3	33.6	33.9	30.4	0.43
	Fish	24.7	22.6	27.1	27.1	0.12	23.8	25.2	24.3	27.7	0.16
	Soybeans and soy product	24.5	24.7	21.0	25.6	0.94	23.0	23.1	22.8	27.5	0.14
	Vegetables	91.6	86.8	91.8	109.5	0.024	84.8	101.6	93.6	124.5	<0.0001
	Fruits	26.7	22.2	22.2	23.3	0.37	23.9	27.8	22.5	29.2	0.37
	Milk and dairy products	134.5	112.9	126.8	118.3	0.49	117.1	118.1	142.0	147.9	0.025
	Sweets and snacks	36.7	40.1	35.1	34.0	0.21	40.9	35.4	38.4	41.2	0.75
	Eggs	22.3	18.9	20.8	20.2	0.54	20.3	18.8	19.8	20.6	0.74

Grades 4–6	Number of respondents	69	73	68	82		62	67	67	70	
	% of correct answers	47.1	63.8	74.7	85.4		48.2	63.5	73.9	86.5	
	Food intakes, adjusted mean,^b^ g/1000 kcal										
	Staple foods^c^	251.5	237.0	245.7	244.8	0.78	253.5	239.2	246.7	238.3	0.39
	Rice	202.7	188.2	206.8	201.1	0.72	199.7	192.0	202.7	190.6	0.72
	Bread	13.8	18.2	13.4	15.2	0.89	18.2	18.5	16.8	16.9	0.50
	Meat	36.2	35.3	35.0	34.2	0.47	33.3	33.7	32.1	35.4	0.61
	Fish	24.4	24.1	23.4	27.7	0.22	23.3	27.5	23.4	27.7	0.31
	Soybeans and soy product	23.1	20.1	22.6	25.9	0.20	18.8	19.8	20.8	26.0	0.027
	Vegetables	72.1	82.1	102.1	103.3	<0.0001	81.0	86.6	91.0	103.5	0.020
	Fruits	23.7	20.6	22.7	23.6	0.85	21.8	22.6	23.7	28.2	0.080
	Milk and dairy products	108.7	141.9	134.6	150.5	0.032	115.1	100.9	119.6	118.6	0.53
	Sweets and snacks	40.5	36.4	35.2	34.3	0.11	41.9	45.5	41.4	39.0	0.34
	Eggs	16.9	20.7	19.9	18.6	0.57	18.1	15.9	19.4	18.3	0.50

a Linear regression analysis was used to test trend of association between knowledge level quartiles and food intakes. Each knowledge quartile was assigned a score: Low = 1, Medium low = 2, Medium high = 3, High = 4. The model included food intake as a dependent variable and knowledge quartile score and grade of the children as independent variables.

b Food intakes adjusted for grade by analysis of covariance were shown.

c Intake of staple foods was sum of rice, bread, and noodle intake.

**Table 4:** Relationship between the percentage of correct answers for the nutrition knowledge questionnaire in guardians of primary school children and dietary intakes in their children.

Analyzed population	Groups by the percentage of correct answers in guardians	Boys				Girls			
		Low	Medium	High	*p* for trend^a^	Low	Medium	High	*p* for trend^a^
Grade 1–3 (*n* = 219)	Number of subjects	35	37	34		40	33	40	
	% correct answers in guardians^b^	58.8	73.5	82.7		59.8	73.4	81.9	
	Food intakes, adjusted mean,^c^ g/1000 kcal								
	Staple foods^d^	245.5	239.3	241.0	0.75	250.6	246.1	220.4	0.060
	Rice	191.9	179.3	191.7	0.99	198.4	201.1	171.0	0.11
	Bread	19.5	23.8	20.4	0.82	15.8	16.6	21.0	0.069
	Meat	30.4	34.0	34.5	0.15	30.4	32.9	31.4	0.75
	Fish	22.2	26.7	26.6	0.17	23.4	29.6	24.0	0.85
	Soybeans and soy product	28.6	23.1	32.4	0.40	20.5	26.0	26.2	0.15
	Vegetables	82.0	99.0	105.9	0.043	79.7	106.4	111.1	0.025
	Fruits	27.4	27.6	26.3	0.81	18.2	32.1	33.6	0.006
	Milk and dairy products	130.3	122.9	128.0	0.91	149.6	128.6	135.4	0.49
	Sweets and snacks	34.8	36.0	33.5	0.76	39.5	34.1	42.9	0.53
	Eggs	19.4	18.4	19.8	0.87	16.1	19.7	19.0	0.21

Grade 4–6 (*n* = 205)	Number of subjects	36	37	38		32	32	30	
	% of correct answers in guardians^b^	58.5	73.6	81.4		60.1	75.2	83.6	
	Food intakes, adjusted mean,^c^ g/1000 kcal								
	Staple foods^d^	238.6	258.7	239.2	0.99	252.4	225.3	225.2	0.10
	Rice	192.6	215.7	193.2	0.99	211.3	180.2	178.5	0.073
	Bread	14.9	16.3	16.7	0.58	15.2	14.5	19.0	0.25
	Meat	30.1	34.4	36.0	0.14	33.1	35.6	36.7	0.42
	Fish	26.9	26.3	24.3	0.52	28.1	26.1	30.7	0.60
	Soybeans and soy product	23.7	22.0	22.0	0.61	22.6	24.1	27.2	0.37
	Vegetables	86.6	92.6	88.1	0.90	94.0	105.3	118.7	0.085
	Fruits	30.7	20.0	23.0	0.22	17.9	28.5	27.4	0.055
	Milk and dairy products	146.2	148.2	166.5	0.42	102.1	103.5	126.3	0.24
	Sweets and snacks	40.7	31.9	32.0	0.14	41.1	44.4	37.6	0.57
	Eggs	20.5	18.3	18.9	0.58	17.7	23.4	16.5	0.76

a Linear regression analysis was used to test trend of association between knowledge level tertiles and food intakes. Each knowledge tertile was assigned a score: Low = 1, Medium = 2, High = 3. The model included food intake as a dependent variable and knowledge tertile score and grade of the children as independent variables.

b The percentage of correct answers in the guardians was calculated after excluding the section about awareness of dietary recommendations.

c Food intakes adjusted for grade by analysis of covariance were shown.

d Intake of staple foods was sum of rice, bread, and noodle intake.

**Table 5:** Relationship between selected food intakes in primary school children and nutrition knowledge of the children and their guardians by linear regression analysis.^a^

Food intake, g/1000 kcal	Independent variables in regression models^b^	Model 1		Model 2		Model 3	
		Regression coefficient	*P* value^c^	Regression coefficient	*P* value^c^	Regression coefficient	*P* value^c^
Staple foods^d^	Children's knowledge^e^	−0.16	0.53	−0.24	0.77	−0.17	0.5
	Guardians' knowledge^f^	−0.65	0.033*	−0.65	0.034*	1.76	0.06
	Children's sex^g^	−7.62	0.26	−11.2	0.76	107.5	0.012*
	Grade	−0.82	0.69	−0.83	0.69	−0.72	0.72
	Guardians' income^h^	−3.18	0.42	−3.18	0.43	−3.83	0.33
	Children's knowledge ^∗^ Children's sex			0.05	0.92		
	Guardians' knowledge ^∗^ Children's sex					−1.6	0.0065*

Vegetable	Children's knowledge^e^	0.6	0.0025*	0.95	0.13	0.6	0.0024*
	Guardians' knowledge^f^	1	<0.0001*	0.99	<0.0001*	0.32	0.66
	Children's sex^g^	9.46	0.069	26	0.36	−22.9	0.49
	Grade	1.08	0.5	1.12	0.48	1.05	0.51
	Guardians' income^h^	1.16	0.71	1.16	0.71	1.35	0.66
	Children's knowledge ^∗^ Children's sex			−0.23	0.56		
	Guardians' knowledge ^∗^ Children's sex					0.45	0.32

Fruit	Children's knowledge^e^	0.13	0.13	−0.24	0.39	0.14	0.12
	Guardians' knowledge^f^	0.06	0.57	0.06	0.54	−0.83	0.0092*
	Children's sex^g^	0.75	0.74	−16.7	0.19	−41.9	0.0042*
	Grade	−0.06	0.94	−0.1	0.89	−0.09	0.89
	Guardians' income^h^	2.4	0.079	2.4	0.079	2.63	0.052
	Children's knowledge ^∗^ Children's sex			0.24	0.16		
	Guardians' knowledge ^∗^ Children's sex					0.59	0.0032*

Sweets and snacks	Children's knowledge^e^	−0.05	0.55	0.25	0.36	−0.05	0.57
	Guardians' knowledge^f^	−0.12	0.24	−0.13	0.23	−0.62	0.055
	Children's sex^g^	5	0.03*	19.3	0.13	−18.6	0.21
	Grade	0.31	0.66	0.35	0.62	0.29	0.68
	Guardians' income^h^	0.33	0.8	0.33	0.81	0.46	0.73
	Children's knowledge ^∗^ Children's sex			−0.2	0.25		
	Guardians' knowledge ^∗^ Children's sex					0.33	0.1

a Among 316 analyzed guardians, 12 did not answer the question about household income, and the pairs of these guardians and their children were deleted from the analysis in Table 5.

b Linear regression model was used to examine the relationship between children's food intake and nutrition knowledge of the children and their guardians. All models include food intake (continuous, g/kcal) as a dependent variable. Model 1: Independent variables were children's knowledge, guardians' knowledge, children's sex, grade, and guardians' income. Model 2: Independent variables were children's knowledge, guardians' knowledge, children's sex, grade, guardians' income, and an interaction term between children's knowledge and children's sex. Model 3: Independent variables were children's knowledge, guardians' knowledge, children's sex, grade, guardians' income, and an interaction term between guardians' knowledge and children's sex.

c Results in tests for regression coefficients, “*” shows *p* < 0.05.

d Intake of staple foods was sum of rice, bread, and noodle.

e Children's knowledge is the percentage of correct answers in nutrition knowledge questionnaire for children, and used as a continuous variable in the models.

f Guardians' knowledge is the percentage of correct answers in nutrition knowledge questionnaire for adults, and used as a continuous variable in the models. The percentage of correct answers in the guardians was calculated after excluding the section about awareness of dietary recommendations.

g Children's sex was coded as below: boy = 1, girl = 2. The reference was the boy.

h Guardians' income was categorized into four groups, and the lowest income group was set as the reference. Each income group was assigned a score: <3 million yen = 1, 3 to <6 million yen = 2, 6 to <9 million yen = 3, and ≥9 million yen = 4. Linear regression analysis was used to test trend of association.

## Discussion

We found that dietary intake in children was associated with nutrition knowledge of children and their guardians. Higher vegetable intake was significantly related with higher knowledge in both child and guardian. As an exception, we saw no significant relationship between vegetable intake in children in higher grades and guardian knowledge. Older children, particularly boys, might have been less compliant and guardian advice about dietary intake might not have been effective. These results were stable even if vegetables were subcategorized by their nutritional characteristics, such as beta-carotene content (data not shown). Although the number of subjects was insufficient to show a significant relationship for many foods, higher children's knowledge might have been associated, for example, with higher intake of fish in both boys and girls.

We used higher intake of fruits, vegetables, soybeans, and fish as indices of “healthy dietary habits” in this study. Similarly, higher intake of sweets and snacks were used as an index of “unhealthy dietary habits”. There are several past studies using “healthy dietary habits” as outcomes. Most of them defined higher intakes of vegetables/fruits and utilization of low-fat milk/snacks were “healthy”, and higher intakes of fat and fried foods were defined as “unhealthy”.^43^, ^44^ Our definition of “heathy dietary habits” is compatible with these previous studies.

Further, our results suggested that relationships between dietary intake and nutrition knowledge differed between boys and girls in some foods. Intake of soybeans and soy products and fruits were higher only in higher-grade girls with higher knowledge level. Significant effect modification by children's sex was observed in the relationship between guardians' nutrition knowledge and fruit intake in children. This result might imply that social pressures for healthy dietary habits differ between boys and girls and might explain the difference in future health outcomes between men and women, at least to some extent.^45^, ^46^ Regarding staple foods, girls with higher knowledge tended to consume less staple foods. Similarly, guardians with higher knowledge tended to provide less staple foods, particularly rice, for their girls only. The effect of guardians' knowledge on children's staple food intake was significantly different between boys and girls. In Japan, since many people believe that a low-carbohydrate diet, such as a diet that includes less rice, is effective for weight loss,^47^, ^48^ it is understandable that girls who aimed to lose weight or guardians having such a daughter would decrease the intake or servings of rice. The proportion of our study children who were trying to lose weight was 27.8% in higher-grade girls, but only 18.8% in higher-grade boys. In addition, there are some studies showing heritability of food preferences in children.^49^, ^50^ Food preferences and behavioral change after acquiring nutrition knowledge might be genetically different between boys and girls, but further studies are needed to clarify these points.

A second important implication of the study is that child's and guardian's nutrition knowledge were both important for the quality of dietary intake in children. Since children are usually provided foods by their guardians, it appears that guardians determine what their children eat. However, child and guardian knowledge might impact children's vegetable intake independently. In the present study, there was no significant correlation between children's nutrition knowledge and guardians' knowledge (data not shown). Guardian nutrition knowledge might slightly affect the dietary intake of the child, possibly through the provision of foods, but communication between guardian and child about nutrition and foods might be insufficient. Räsänen et al. also reported that when a nutrition counseling intervention was given to parents, the food intake of the children was improved in the intervention group compared with the control group, but that the nutrition knowledge score of children was not improved in the intervention group.^7^ Food education that targets both child and guardian might be more effective than programs aimed at either child or guardian separately.

Several studies have examined the relationship between nutrition knowledge and dietary intake in adults, mostly in western countries. Regarding children's knowledge and their intake, only a few studies have been reported.^12^, ^51^ This type of study might be difficult to conduct because measurement tools for both nutrition knowledge and dietary intake in children are insufficient. In contrast, a number of studies have examined the relationship between the dietary intake of children and the nutrition knowledge of their guardians, particularly mothers^28^, ^52^, ^53^, ^54^: most suggested that higher maternal nutrition knowledge promoted a healthy dietary intake in children. In contrast, Williams et al. reported a significant association between maternal nutrition knowledge and maternal diet but no association between maternal nutrition knowledge and children's diet.^53^ It is possible that the nutrition knowledge of children also play an important role in child's dietary intake. Children might be able to alter variety of foods frequently offered from their guardians by refusing foods they would not like to consume.

Our study has several strengths. First, the data included information about both the child's and guardians' nutrition knowledge, allowing analysis of their combined effect. Second, volunteer bias in the participating children was probably low because most pupils in the four primary schools answered the questionnaires as a part of food education program and the participation rate was high (62.8%). Third, the nutrition knowledge questionnaires for this study were developed based on several previously validated questionnaires, and questions and wording were carefully selected after a pilot study. The Cronbach's alpha (after exclusion of the section about awareness of dietary recommendations) showed internal consistency between the sections, with scores of 0.66, 0.76, and 0.69 in lower graders, higher graders, and adults, respectively. Usually, a Cronbach's alpha >0.7 is considered to indicate sufficient internal consistency.^55^ Although two coefficients were slightly below this level, there were only five knowledge sections for lower graders and three (four before exclusion) for adults, and each section measures different aspects of knowledge about nutrition and related information. The internal consistency of the questionnaires was therefore considered reasonable. Last, dietary intake of major foods was assessed quantitatively using the BDHQ15y.

Several limitations of the study should also be noted. First, although more than 1000 pupils participated, this number was still insufficient because stratified analysis was performed. The participation rate of guardians was quite low, raising the possibility that participants were highly health-conscious. This might have caused the non-significant relationship between the guardians' nutrition knowledge and children's dietary intake. Conversely, we consider that the relationship between the vegetable intake of children and guardians' knowledge is highly significant. Second, validation of the BDHQ15y is not complete. One validation study showed significant correlations between nutrient intake estimates using the BDHQ15y and biomarkers for α-carotene, β-carotene, β-cryptoxanthin, eicosapentaenoic acid, and docosahexaenoic acid,^40^ and it indicated that estimates of vegetables and fish intake are reasonably precise. Further, given that the BDHQ for adults showed sufficient validity^36^, ^37^ and the BDHQ15y uses almost the same nutritional calculation algorithm as the BDHQ, subject ranking by intake amount was estimated correctly even if the absolute values of estimated intake were biased to some extent. Last, the study was conducted in a single town in Okinawa, Japan. The generalizability of the results should be carefully considered because commonly consumed foods and food availability differ between areas and countries.

In conclusion, we developed nutrition knowledge questionnaires for primary school children and adults in Japan. A higher level of child and guardian nutrition knowledge was associated with healthy dietary habits in the children, such as higher vegetable intake. The impact of sex differences on the effect of nutrition knowledge should be investigated further in the future.

## Conflicts of interest

None declared.
