# Supplementary material for: Relationship between nutrition knowledge and dietary intake among primary school children in Japan: Combined effect of children's and their guardians' knowledge
Source: J Epidemiol. 2017 May 30;27(10):483–91. doi: 10.1016/j.je.2016.09.014 (PMC5602805; doi:10.1016/j.je.2016.09.014)
Supplement: Supplementary file 1 [file mmc1.pdf]

eTable 1. Items in the Nutrition Knowledge Questionnaire used for determining the nutrition knowledge score

Target group	Knowledge section	Question	No.	Choice (for reference, correct answers are marked with a circle)
Pupils in grades 1-3	Categorization of foods based on nutritional similarity	Circle the item number of one food which is different from other foods in a group of four foods.	1	cabbage, cucumber, Japanese radish, white rice(○)
			2	apple, tomato(○), satsuma mandarine, strawberry
			3	yogurt, milk, margarine(○), cheese
			4	broccoli, pumpkin, shiitake mushroom(○), sweet pepper
			5	banana(○), potato chips, chocolate, custard pudding
			6	ham, bacon, sausage, canned tuna(○)
	Knowledge about foods as nutrient sources	Which food contains more salt?	1	chikuwa[a](○), tofu[b]
			2	cucumber, takuan[c](○)
			3	pork, sausage(○)
			4	raw tuna, ikura[d](○)
			5	biscuit(○), caramel
		Which food contains more fat?	6	cake(○), drop
			7	egg(○), onion
			8	orange juice, ice cream(○)
			9	white rice, white table bread(○)
			10	peanut(○), baked sweet potato
	Physiological function of nutrients in human body	Choose two foods which mainly work as energy (heat and power) in the human body.	1	rice(○), lettuce, egg, spaghetti(○), string beans
		Choose two foods which mainly constitutes blood and muscles in the human body.	2	sweet pepper, sardine(○), pork(○), white sugar, butter
		Choose two foods which mainly help regulate physical condition in the human body.	3	tomato(○), bread, milk, spinach(○), tofu[b]
	Relationship between nutrients and health outcomes	Choose all foods which should be consumed more to become healthy	1	carrot(○), French fries, milk(○), wakame[e](○), doughnut, white sugar, chocolate, tofu[b](○), mayonnaise, apple(○)
	Others 1 (e.g. dietary behavior, food choice)	Drinking milk makes your bones and teeth stronger.	1	correct(○), wrong
		When you are busy, it is fine to eat only two meals a day.	2	correct, wrong(○)
		Children don't have to eat vegetables as long as they eat enough rice and meat for their growth.	3	correct, wrong(○)
		There is a correlation between what you eat and the level of your energy for playing, studying, etc.	4	correct(○), wrong
		It is the best for you to flavor foods as you like	5	correct, wrong(○)
		I had cookies for a snack instead of an apple, which should help me stay healthy and energetic.	6	correct, wrong(○)
Pupils in grades 4-6	Knowledge about foods as nutrient sources	Each group of four foods below includes one with a different principal nutrient from the others. Circle the item number of one food which is different.	1	white rice, sardine(○), sweet corn, potato
			2	soybeans, pumpkin(○), cheese, pork (lean cut)
			3	butter, mayonnaise, honey(○), salad oil
		Circle the item numbers of three foods which should be consumed when you need calcium.	4	buckwheat noodles, tuna, cheese(○), potato, shirasuboshi[f](○), soybeans(○)
		Circle the item numbers of three foods which should be consumed when you need vitamin C.	5	kiwifruit(○), sweet pepper(○), bread, milk, broccoli(○), chicken
		Circle the item numbers of three foods which contain a lot of salt.	6	dried horse mackerel(○), plain spaghetti, takuan[c](○), daifuku-mochi[g], lemon, sausage(○), liver, shrimp
		Circle the item numbers of three foods which contain a lot of fat.	7	lettuce, margarine(○), rice cake, sesame(○), eggplant, egg yolk, potato(○), chicken tenderloin
	Physiological function of nutrients in human body	The nutrients which mainly work as a source of energy (e.g. power for physical activity) are protein, fat, and carbohydrate.	1	correct(○), wrong, not sure
		The foods which mainly work as a source of energy (e.g. power for physical activity) include meat, fish, egg, fat and oil (butter, vegetable oil, etc,), and rice.	2	correct(○), wrong, not sure
		The unit of energy is kilocalorie (kcal).	3	correct(○), wrong, not sure
		The nutrient which mainly constitutes blood and muscles is carbohydrate.	4	correct, wrong(○), not sure
		Since fat harms your health, it is better to minimize its intake. In fact, there is no need to consume fat at all.	5	correct, wrong(○), not sure
		Fish oil and fat on meat have the same characteristics.	6	correct, wrong(○), not sure

		You should eat a lot of dietary fiber which is contained mainly in vegetables, pulses, and seaweeds, because it is good for your health.	7	correct(○), wrong, not sure
		Vitamins help regulate physical condition	8	correct(○), wrong, not sure
		Calcium is a main component of muscles.	9	correct, wrong(○), not sure
		Since salt is essential to human life, consumption of a lot of salt is good for your health.	10	correct, wrong(○), not sure
	Relationship between nutrients and health outcomes	What you eat affects the condition of your health.	1	correct(○), wrong, not sure
		Excess energy intake from meals does not cause health problems in children.	2	correct, wrong(○), not sure
		Excess intake of fats and oils can cause heart diseases.	3	correct(○), wrong, not sure
		Excess intake of salty foods can cause blood vessel disorders in the brain.	4	correct(○), wrong, not sure
		Vitamin deficiency does not cause death.	5	correct, wrong(○), not sure
		Calcium deficiency can cause bone fracture, particularly late in life.	6	correct(○), wrong, not sure
		Excess intake of dietary fiber can cause cancer.	7	correct, wrong(○), not sure
		People who are thinner or fatter than average tend to get sick more easily.	8	correct(○), wrong, not sure
		Excess intake of sugar or sugary foods/drinks can cause obesity and dental cavities.	9	correct(○), wrong, not sure
		A diet consisting only of fruit and vegetables is better than that consisting of more diverse foods.	10	correct, wrong(○), not sure
Adults	Knowledge about foods as nutrient sources	Are foods listed below rich in starch?		
		cheese	1	rich, poor(○), not sure
		butter	2	rich, poor(○), not sure
		nuts	3	rich, poor(○), not sure
		white rice	4	rich(○), poor, not sure
		cornflakes	5	rich(○), poor, not sure
		Are foods listed below rich in protein?		
		chicken	6	rich(○), poor, not sure
		cheese	7	rich(○), poor, not sure
		boiled beans	8	rich(○), poor, not sure
		butter	9	rich, poor(○), not sure
		cream	10	rich, poor(○), not sure
		Are foods listed below rich in fats and oils?		
		pasta (without sauce)	11	rich, poor(○), not sure
		boiled beans	12	rich, poor(○), not sure
		sausage	13	rich(○), poor, not sure
		honey	14	rich, poor(○), not sure
		nuts	15	rich(○), poor, not sure
		French bread	16	rich, poor(○), not sure
		Are foods listed below rich in dietary fiber?		
		cornflakes	17	rich(○), poor, not sure
		banana	18	rich(○), poor, not sure
		lean meats	19	rich, poor(○), not sure
		broccoli	20	rich(○), poor, not sure
		fish	21	rich, poor(○), not sure
		boiled beans	22	rich(○), poor, not sure
		Are foods listed below rich in vitamin C?		
		white rice	23	rich, poor(○), not sure
		sweet potato	24	rich(○), poor, not sure
		natto[h]	25	rich, poor(○), not sure
		broccoli	26	rich(○), poor, not sure
		butter	27	rich, poor(○), not sure
		Are foods listed below rich in calcium?		
		momen-tofu[i]	28	rich(○), poor, not sure
		chicken	29	rich, poor(○), not sure
		tuna	30	rich, poor(○), not sure

	hijiki[j]	31	rich(○), poor, not sure
	Japanese mustard spinach	32	rich(○), poor, not sure
	Are foods listed below rich in salt?		
	sausage	33	rich(○), poor, not sure
	white rice	34	rich, poor(○), not sure
	lean meats	35	rich, poor(○), not sure
	frozen vegetables	36	rich, poor(○), not sure
	cheese	37	rich(○), poor, not sure
	Are foods listed below rich in sugar?		
	banana	38	rich, poor(○), not sure
	ice cream	39	rich(○), poor, not sure
	soda pop	40	rich(○), poor, not sure
	tomato ketchup	41	rich(○), poor, not sure
	canned fruit (with syrup)	42	rich(○), poor, not sure
Physiological function of nutrients in human body	Carbohydrates are a major source of energy in our meals.	1	correct(○), wrong, not sure
	Excess intake of fats and oils is the only cause of obesity.	2	correct, wrong(○), not sure
	Fats and oils are harmful to our health, and their intake should be avoided as much as possible.	3	correct, wrong(○), not sure
	There are some high-fat foods that contain no cholesterol.	4	correct(○), wrong, not sure
	Adults have no need to consume protein as long as they get sufficient energy from fats and carbohydrates.	5	correct, wrong(○), not sure
	Which nutrient contains the highest energy (so-called "calorie") per unit weight?	6	protein, fat(○), starch, dietary fiber
	Dietary fiber is a type of carbohydrate, and functions as an important energy source.	7	correct, wrong(○), not sure
	The only beneficial health effect of fruit and vegetables is that they supply vitamins and minerals to our body.	8	correct, wrong(○), not sure
	Vitamins are either water-soluble or fat-soluble. Both types of vitamins should be taken as much as possible in any form, including dietary supplements.	9	correct, wrong(○), not sure
	Vitamin deficiency can cause death.	10	correct(○), wrong, not sure
	When calcium intake is insufficient, the bones release calcium to maintain the blood calcium level.	11	correct(○), wrong, not sure
	What vitamine is necessary for effective use of calcium in human body?	12	Vitamin A, Vitamin C, Vitamin D(○), Vitamin K
	The major mineral which can be obtained from salt is sodium.	13	correct(○), wrong, not sure
	Salt is essential for human life, and we need to regularly consume a certain quantity of salt.	14	correct(○), wrong, not sure
	Sugar is a type of carbohydrate, and it can substitute starch as a major energy source.	15	correct, wrong(○), not sure
Awareness of dietary recommendations[k]	Balanced diet means eating equal amounts of different foods.	1	correct, wrong(○), not sure
	How much energy do healthy adults (aged 18-69) need per day to perform ordinary physical activities, assuming they are not extremely lethargic, and are neither hard physical laborers nor athletes?	2	around 2000kcal for men, around 1600kcal for women<br>around 2500kcal for men, around 2000kcal for women(○)<br>around 3000kcal for men, around 2500kcal for women<br>around 3500kcal for men, around 3000kcal for women
	According to the recommendation of the Ministry of Health, Labour and Welfare, what is the recommended percentage of daily energy (or calorie) intake from fats? (Since the recommended values are different by age, the choices are presented as ranges of values.)	3	5 - 10 or 15%<br>10 - 15 or 20%<br>20 - 25 or 30%(○)<br>30 - 35 or 40%
	According to the recommendation of the Ministry of Health, Labour and Welfare, how much salt intake (gram per day) is recommended for Japanese adults (more than 18 years old)?	4	<5 g for men, <4.5 g for women<br><9 g for men, <7.5 g for women(○)<br><12 g for men, <10 g for women<br><15 g for men, <12 g for women
	According to the recommendation of the Ministry of Health, Labour and Welfare, how much calcium intake (miligrams per day) is recommended for Japanese adults (aged 18 or older)? As the recommended values differ by age, choose one that most closely approximates the correct values.	5	≥500 mg for men, ≥ 450 mg for women<br>≥600 mg for men, ≥ 550 mg for women<br>≥700 mg for men, ≥ 650 mg for women(○)<br>≥800 mg for men, ≥ 750 mg for women
Relationship between nutrients and health outcomes	Do you think that risk of a certain cancer can be decreased by		

actions listed below?	
consume more dietary fiber	1 decrease(○), not decrease, not sure
consume less sugar	2 decrease, not decrease(○), not sure
consume less fruit	3 decrease, not decrease(○), not sure
consume less salt	4 decrease(○), not decrease, not sure
consume more fruit and vegetables	5 decrease(○), not decrease, not sure
consume less artificial preservatives and food additives	6 decrease(○), not decrease, not sure
Do you think that heart diseases (in particular, cardiovascular diseases such as angina pectoris and myocardial infarction) can be prevented by actions listed below?	
consume more dietary fiber	1 prevented(○), not prevented, not sure
consume less saturated fatty acid	2 prevented(○), not prevented, not sure
consume less polyunsaturated fatty acid	3 prevented, not prevented(○), not sure
consume less salt	4 prevented(○), not prevented, not sure
consume more fruit and vegetables	5 prevented(○), not prevented, not sure
consume less artificial preservatives and food additives	6 prevented(○), not prevented, not sure
Do you think the health problems and diseases listed below can be prevented by sufficient intake of dietary fiber?	
constipation	1 prevented(○), not prevented, not sure
stomach cancer	2 prevented, not prevented(○), not sure
obesity	3 prevented(○), not prevented, not sure
gout	4 prevented, not prevented(○), not sure
myocardial infarction	5 prevented(○), not prevented, not sure
Do you think the health problems and diseases listed below can be prevented by reducing salt intake?	
hypertension	1 prevented(○), not prevented, not sure
stroke (cerebral hemorrhage, cerebral infarction)	2 prevented(○), not prevented, not sure
stomach cancer	3 prevented(○), not prevented, not sure
bone fracture	4 prevented, not prevented(○), not sure
hepatitis	5 prevented, not prevented(○), not sure

The original questionnaires are in Japanese.

[a] "chikuwa" is tube-like fish paste products.

[b] "tofu" is soybean curd.

[c] "takuan" is pickled Japanese raddish.

[d] "ikura" is salmon roe (usually salted for preservation).

[e] "wakame" is a kind of seaweed.

[f] "shirasuboshi" is dried young sardines (usually salted for preservation).

[g] "daifuku-mochi" is a Japanese confection consisting of a small round rice cake stuffed with sweetened red bean paste.

[h] "natto" is fermented soybeans.

[i] "momen-tofu" is a type of tofu (firm tofu).

[j] "hijiki" is a kind of seaweed.

[k] The questions for adults were compiled based on the Dietary Reference Intakes for Japanese (DRI), 2010.
The DRI 2015 had not yet been released when the questionaire survey was conducted.
